# The Influence of Environmental Conditions on Pacing in Age Group Marathoners Competing in the “New York City Marathon”

**DOI:** 10.3389/fphys.2022.842935

**Published:** 2022-06-14

**Authors:** Katja Weiss, David Valero, Elias Villiger, Volker Scheer, Mabliny Thuany, Ivan Cuk, Thomas Rosemann, Beat Knechtle

**Affiliations:** ^1^ Medbase St. Gallen Am Vadianplatz, St. Gallen, Switzerland; ^2^ Ultra Sports Science Foundation, Pierre-Benite, France; ^3^ Klinik für Allgemeine Innere Medizin, Kantonsspital St. Gallen, St. Gallen, Switzerland; ^4^ Centre of Research, Education Innovation and Intervention in Sport (CIFI2D), Faculty of Sport, University of Porto, Porto, Portugal; ^5^ Faculty of Physical Education and Sports Management, Singidunum University, Belgrade, Serbia; ^6^ Institute of Primary Care, University of Zurich, Zurich, Switzerland

**Keywords:** marathon, running, master, temperature, sunshine

## Abstract

**Background:** The two aspects of the influence of environmental conditions on marathon running performance and pacing during a marathon have been separately and widely investigated. The influence of environmental conditions on the pacing of age group marathoners has, however, not been considered yet.

**Objective:** The aim of the present study was to investigate the association between environmental conditions (i.e., temperature, barometric pressure, humidity, precipitation, sunshine, and cloud cover), gender and pacing of age group marathoners in the “New York City Marathon”.

**Methodology:** Between 1999 and 2019, a total of 830,255 finishes (526,500 males and 303,755 females) were recorded. Time-adjusted averages of weather conditions for temperature, barometric pressure, humidity, and sunshine duration during the race were correlated with running speed in 5 km-intervals for age group runners in 10 years-intervals.

**Results:** The running speed decreased with increasing temperatures in athletes of age groups 20–59 with a pronounced negative effect for men aged 30–64 years and women aged 40–64 years. Higher levels of humidity were associated with faster running speeds for both sexes. Sunshine duration and barometric pressure showed no association with running speed.

**Conclusion:** In summary, temperature and humidity affect pacing in age group marathoners differently. Specifically, increasing temperature slowed down runners of both sexes aged between 20 and 59 years, whereas increasing humidity slowed down runners of <20 and >80 years old.

## Introduction

The “New York City Marathon” is held since 1970 and is considered the largest city marathon in the world, with tens of thousands of elite and recreational marathoners participating each year (https://www.nyrr.org/races/2021tcsnewyorkcitymarathon). Since the first edition, the overall participation increased across calendar years with a more pronounced participation in women, resulting in a decrease of the men-to-women ratio (Vitti et al., 2020). Over the years, the average age of the successful finishers increased as did their overall race times (Vitti et al., 2020).

A half-century of history highlighted the search for better performance among the athletes, as knowledge about performance predictors among the researchers. In this sense, runners’ profile, performance predictors related individual, and environment have been studied (Nikolaidis et al., 2018; Knechtle et al., 2021). It is well-known that environmental conditions have a strong influence on marathon running performance (Trapasso and Cooper, 1989; Zhang et al., 1992; Ely et al., 2007; Vihma, 2010; El Helou et al., 2012; Knechtle et al., 2021; Mantzios et al., 2021; Trubee et al., 2014; Ely et al., 2008). These influences have been investigated in different large city marathons such as the “Boston Marathon” (Roberts, 2007; El Helou et al., 2012; Knechtle et al., 2019; Nikolaidis et al., 2019; Trapasso and Cooper, 1989), the “Chicago Marathon” (El Helou et al., 2012; Trubee et al., 2014), the “New York City Marathon” (El Helou et al., 2012; Gasparetto et al., 2020; Knechtle et al., 2021), the “Berlin Marathon” (El Helou et al., 2012; Knechtle et al., 2021; Knechtle et al., 2021; Scheer et al., 2021), the “Stockholm Marathon” (Vihma, 2010), the “London Marathon” (El Helou et al., 2012), the “Beijing Marathon” (Zhang et al., 1992), and the “Paris Marathon” (El Helou et al., 2012).

Among the investigated weather variables, the temperature had the highest influence on marathon running performance (Trapasso and Cooper, 1989; Ely et al., 2007; Vihma, 2010; El Helou et al., 2012; Knechtle et al., 2021; Mantzios et al., 2021; Gasparetto et al., 2020; Trubee et al., 2014; Ely et al., 2008) where slower runners slowed down more with increasing temperatures than faster runners (Ely et al., 2007; Ely et al., 2008; Vihma, 2010). Also, in the “New York City Marathon,” high temperatures showed the greatest effect on race times (El Helou et al., 2012; Gasparetto et al., 2020; Knechtle et al., 2021) where higher temperatures mainly affected race times of middle-aged runners (*i.e.,* men of 30–64 years old and women of 40–64 years old) (Knechtle, McGrath et al., 2021).

Taking into account that performance is a complex system, environmental characteristics can influence in decision-making of runners during the running (Renfree et al., 2018). Running speed can be treated as an outcome, part of the relationship among individual, environmental and task, that is a self-regulated during the race event (Newell, 1986). Previous results have shown that the pacing strategy has an influence on marathon race time (Haney et al., 2011; March et al., 2011; Deaner et al., 2015; Breen et al., 2018; Díaz et al., 2019) with differences between race courses (Díaz et al., 2019; Oficial-Casado et al., 2021), between elite marathoners (Oficial-Casado et al., 2021; Díaz et al., 2019; Díaz et al., 2018) and recreational age group runners (Haney et al., 2011; Nikolaidis et al., 2019; Oficial-Casado et al., 2021) and between female and male marathoners (Deaner et al., 2015; Nikolaidis and Knechtle, 2018; Díaz et al., 2019; Muñoz-Pérez et al., 2020). In the “New York City Marathon,” both female and male age group marathoners continuously reduced their running speed during the race with a final sprint in the last segment (Nikolaidis and Knechtle, 2018).

Differences in pacing seemed to exist in the ‘New York City Marathon’ regarding age group runners (Nikolaidis and Knechtle, 2017; Nikolaidis and Knechtle, 2019). When older and younger runners of similar performance were compared, older runners paced with smaller changes in running speed than younger runners (Nikolaidis and Knechtle, 2017). Slower runners showed the largest decrease in running speed at 5, 10, 15, and 20 km but the largest increase in running speed at 35 and 40 km. Faster runners, however, had the least decrease in running speed during the race and the least increase at 40 km (Nikolaidis and Knechtle, 2019).

Actually, we know from the “New York City Marathon” that higher temperatures had a higher effect on older runners and older runners paced differently than younger runners. We have, however, no knowledge about the influence of environmental conditions such as temperature on pacing in age group runners. Therefore, the aim of the present study was to investigate a potential association between environmental conditions such as temperature and running speed for age group marathoners competing in the “New York City Marathon”. We hypothesized that increasing temperatures during race day would have a higher impact on slower (and older) than on faster (and younger) runners.

## Materials and Methods

### Ethical Approval

The institutional review board of St Gallen, Switzerland, approved this study (EKSG 01/06/2010). Since the study involved the analysis of publicly available data, the requirement for informed consent was waived.

### The Race

The “New York City Marathon” is held since 1970 and in addition to the “Boston Marathon” and the “Chicago Marathon,” it is one of the most important and largest running events in the United States of America. The “New York City Marathon” is the marathon with the largest number of participants in the world. The race takes place on the first Sunday in November in New York City. The course is a point-to-point racecourse, that starts on Fort Wadsworth on Staten Island, goes via Brooklyn, Queens and the Bronx and finishes at Central Park in Manhattan. Due to the large number of participants, the start is now in waves, first at 08:00 a.m. with professional wheelchair division, at 08:22 a.m. the handcycle category and selected athletes with disabilities, at 08:40 a.m. the professional women, at 09:05 a.m. the professional men, at 09:10 a.m. the first wave, at 09:55 a.m. the second wave, at 10:40 a.m. the third wave, at 11:20 a.m. the fourth wave and at 12:00 the fifth wave. In 2012, the ‘New York City Marathon’ was not held due to Hurricane Sandy. Therefore, no data for this year exist in our analysis. In 2020 the “New York City Marathon” had to be canceled again as there were significant safety and health concerns for athletes, volunteers and spectators as a result of the COVID-19 pandemic (https://www.nyrr.org/tcsnycmarathon; Scheer et al., 2021).

### Subjects

Data on all successful female and male finishers with first and last name, sex, age, calendar year, split times at 5, 10, 15, 20, 25, 30, 35, 40 km and finish was obtained from the race website (https://www.nyrr.org/tcsnycmarathon).

### Weather Data

Historic weather data in hourly intervals were obtained from Weather History Download New York (https://www.meteoblue.com/en/weather/archive/export/new- york_united-states-of-america_5158128). The weather values available are hourly readings (between 09:00 a.m. and 04:00 p.m.) of the following magnitudes: Temperature (°Celsius), barometric pressure (hPa), humidity (%) and sunshine duration (min). Between 1999 and 2019, no rain has been ever recorded during the “New York City Marathon”. For that reason, the variable rain is not included in our data analysis.

### Data Processing

The processing of the data files involved several steps. First of all, the data was cleaned up and its integrity verified. Then all runners’ records were classified into finish time categories, as follows: less than 2:30 h:min, 2:30 h:min to 3:00 h:min, 3:00 h:min to 3:30 h:min, 3:30 h:min to 4:00 h:min, 4:00 h:min to 4:30 h:min, 4:30 h:min to 5:00 h:min, 5:00 h:min to 5:30 h:min, 5:30 h:min to 6:00 h:min, and over 6:00 h:min. Next, the inclusion of the weather factors to the runners’ records was performed. This was done in two different manners: First using the full race average values and second using the time-adjusted average values. The second method proved to be more revealing. Since the duration of each runner’s race is different, from just over 2 h for elite runners through to 6 or 8 h and over for more recreational runners, the average values of the temperature, pressure, and other weather factors they experience during the race is also slightly different. Taking into account these differences when calculating and imputing the weather values to each record, we were able to better represent the actual average values during their running time. Finally, runners’ records were also categorized into weather categories, in an attempt to magnify any existing associations between the running speed (pace) and the weather factors. To accomplish this, the race records were categorized into “high” and “low” meaning above or below the median (50% percentile) of each weather variable. The median was chosen as the category threshold value as it would yield a similar number of records into each category. After all, that processing was complete, descriptive statistical methods were used to make comparisons between groups and draw insights and conclusions. All data processing was carried out with Python in a Google Colab notebook.

### Statistical Analysis

Descriptive statistical analysis was carried out, including Pearson (r) and Spearman (*ρ*) correlation analysis and the calculation of statistical parameters of the full and partial race times, and the weather factors, of each time-group and each weather category. The resulting values are then presented in tables and charts in terms of their average value (mean) and standard deviation (std), along with maximum (max) and minimum (min) values for each category. The column named “N” represents the number of samples in that specific category. Gaussian (normal) distribution of race times and paces was verified by plotting histograms and statistical significance tests and calculation of *p*-values was done when needed. All analyses were carried out using the Python programming language (Python Software Foundation, https://www.python.org/), in a Google Colab notebook (https://colab.research.google.com/).

## Results

### Runners’ Participation and Running Speed

**FIGURE 1:**
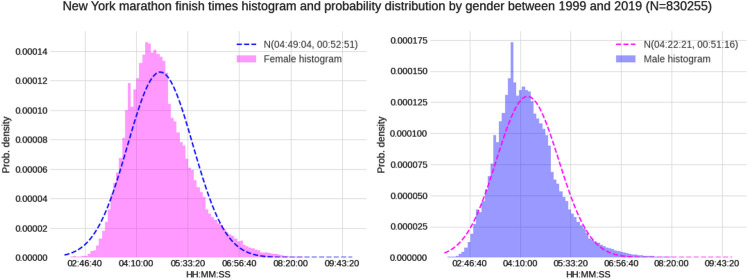
Histograms of female and male race times in the “New York City Marathon”.

**FIGURE 2:**
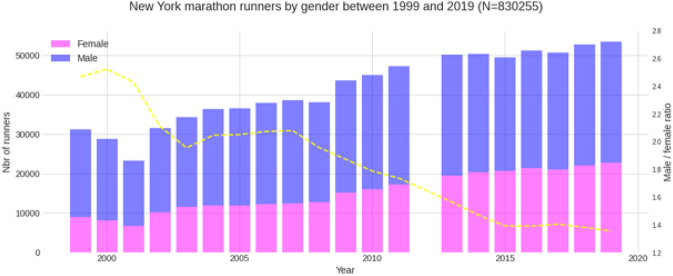
Trend of female and male runners and the male-to-female ration.

**FIGURE 3:**
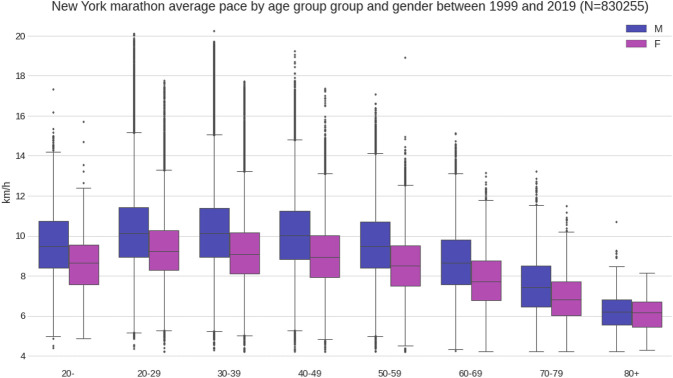
Running speed by age group in 10-years-intervals.

**FIGURE 4:**
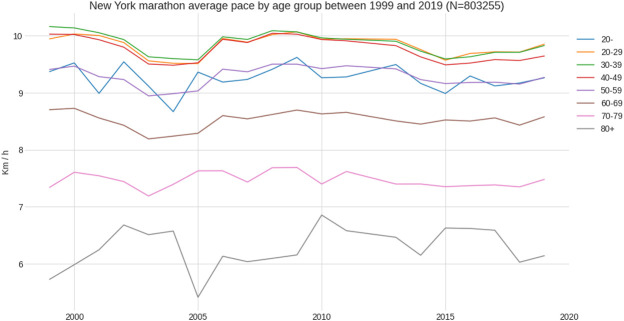
Average running speed by age group between 1999 and 2019.

Between 1999 and 2019, a total of 830,255 finishes (526,500 males and 303,755 females) were recorded. Female mean race time was 04:49:04 (±00:52:51) h:min:s, and male mean race time 04:22:21 (±00:51:16) h:min:s (Figure 1). Across the years, the number of female and male runners increased where the male-to-female ratio decreased (Figure 2). The fastest race times are accomplished by runners in their twenties, thirties and forties (age groups 20–49 years) with a steady decline in running speed from 50 years onwards (Figure 3). Also, across years, the fastest runners were in age groups 20–29 years, 30–39 years and 40–49 years (Figure 4), represented by the three lines at the top of the chart. Just below these age groups, the average running speed curves for the under 20 years (age group -20 years) and 50 years and older (age group 50–59 years) were running close together. This shows that the marathon running performance of runners in age groups 50–59 years and under 20 years has been similar through the years. Further down, dropping about 1 km/h per decade, are the sixties, seventies and over eighty categories.

### Environmental Characteristics and Running Speed

**FIGURE 5:**
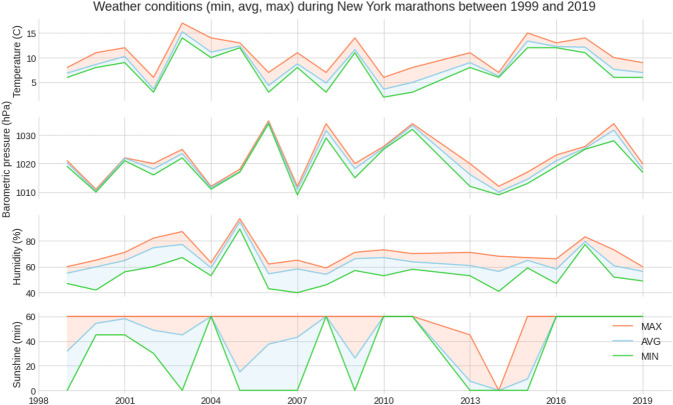
Weather conditions in the “New York City Marathon” between 1999 and 2019.

**FIGURE 6:**
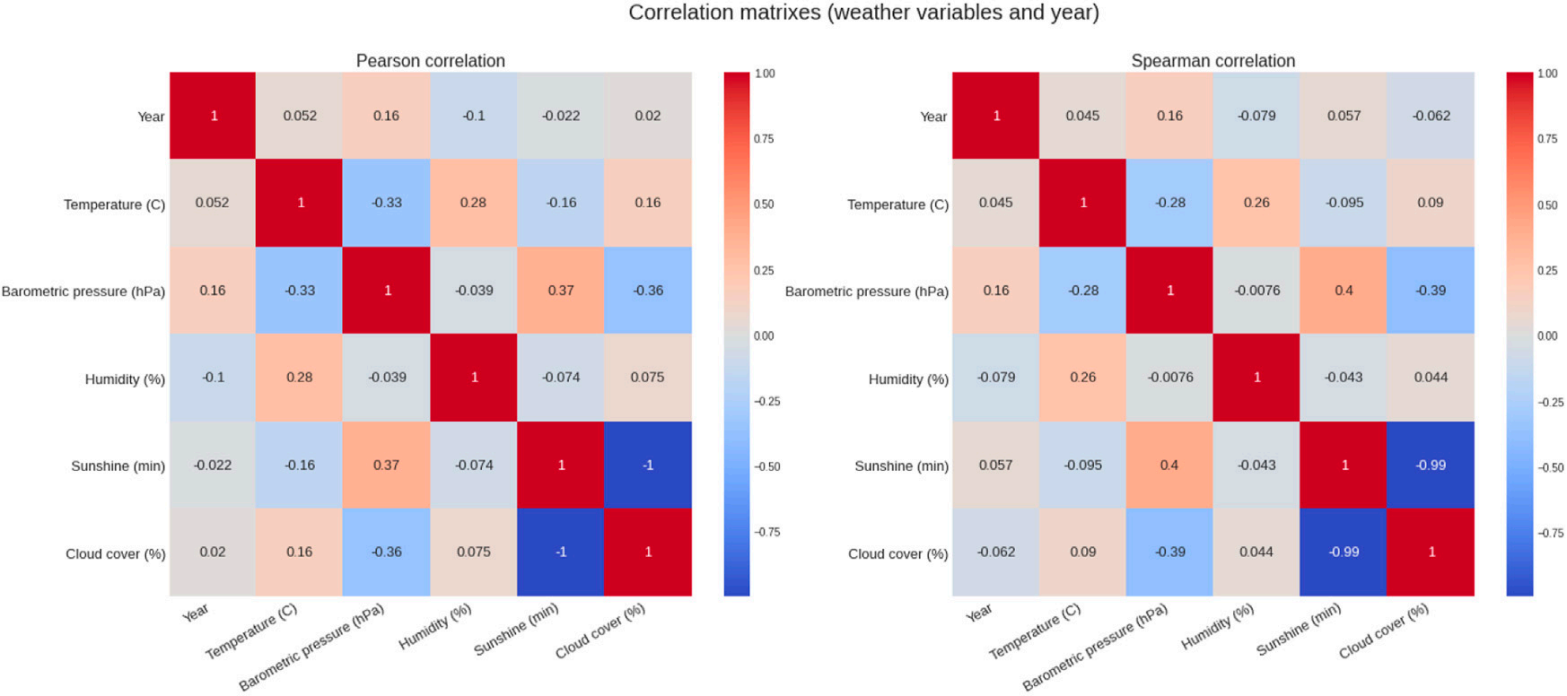
Correlation matrixes for weather variables and year.

**FIGURE 7:**
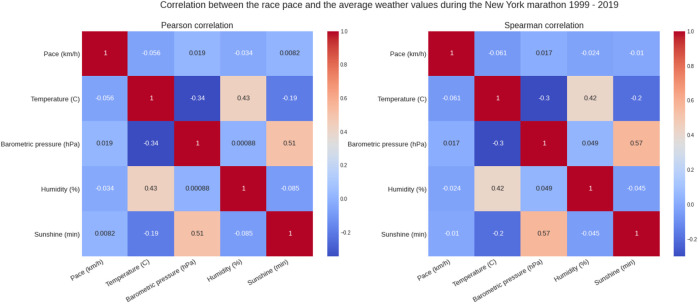
Correlation between running speed and average weather variables.

**FIGURE 8:**
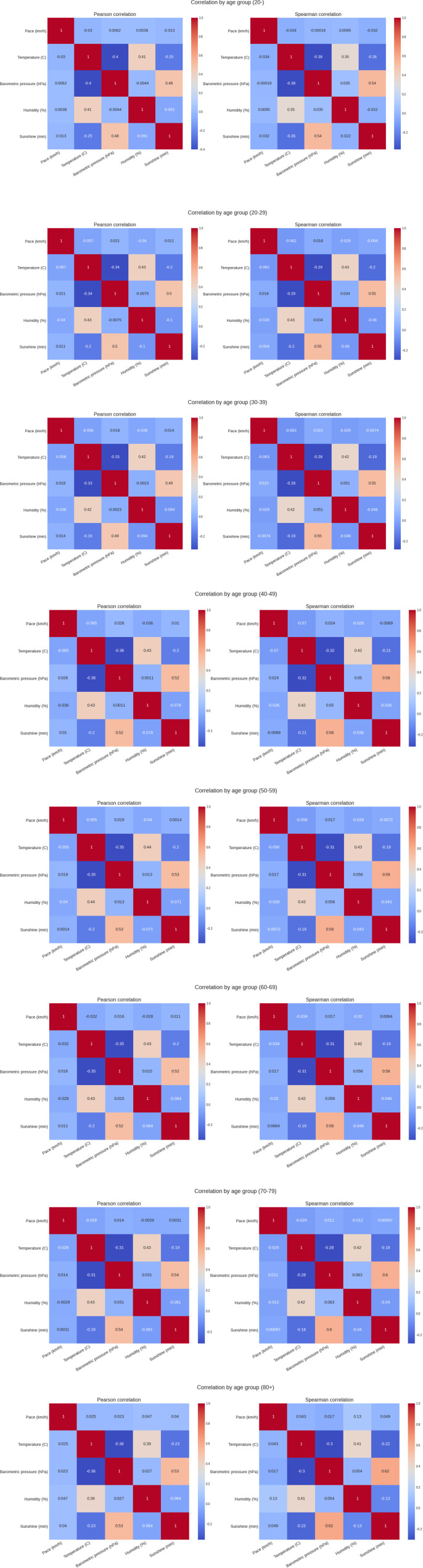
Correlation between running speed and average weather variables by age groups.

**FIGURE 9:**
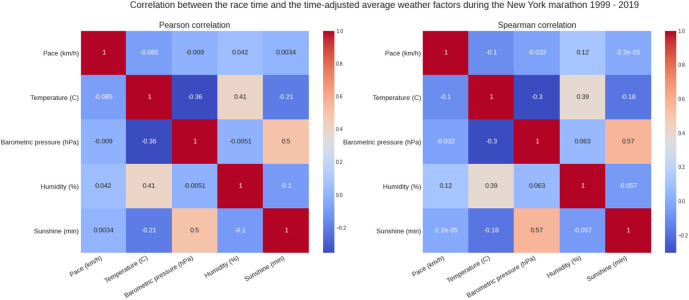
Correlation between race times and time-adjusted weather variables.

**FIGURE 10:**
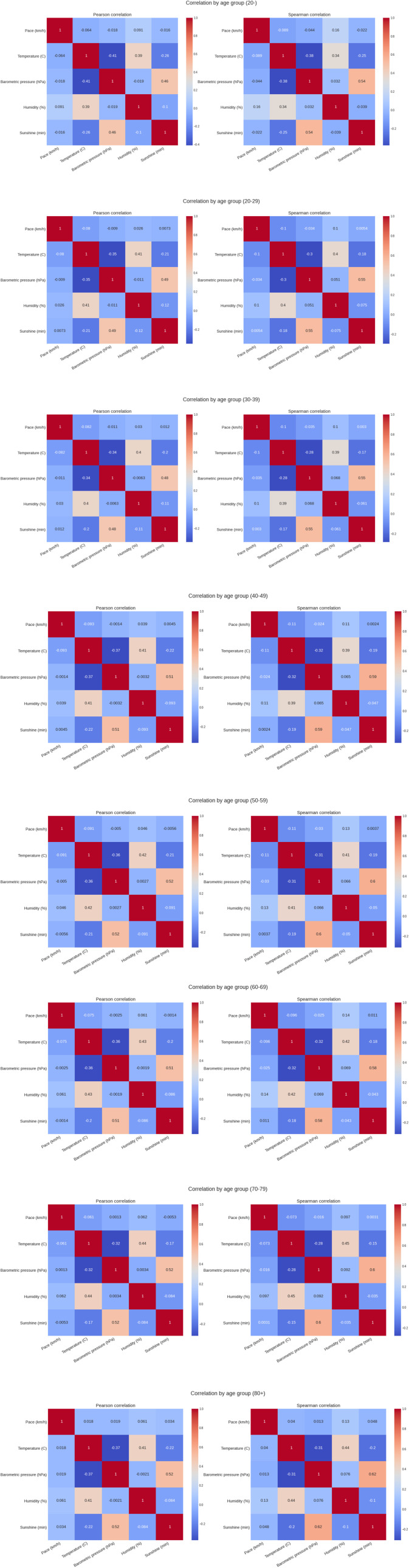
Correlation analyses using time‐adjusted average of weather conditions by age groups

**FIGURE 11:**
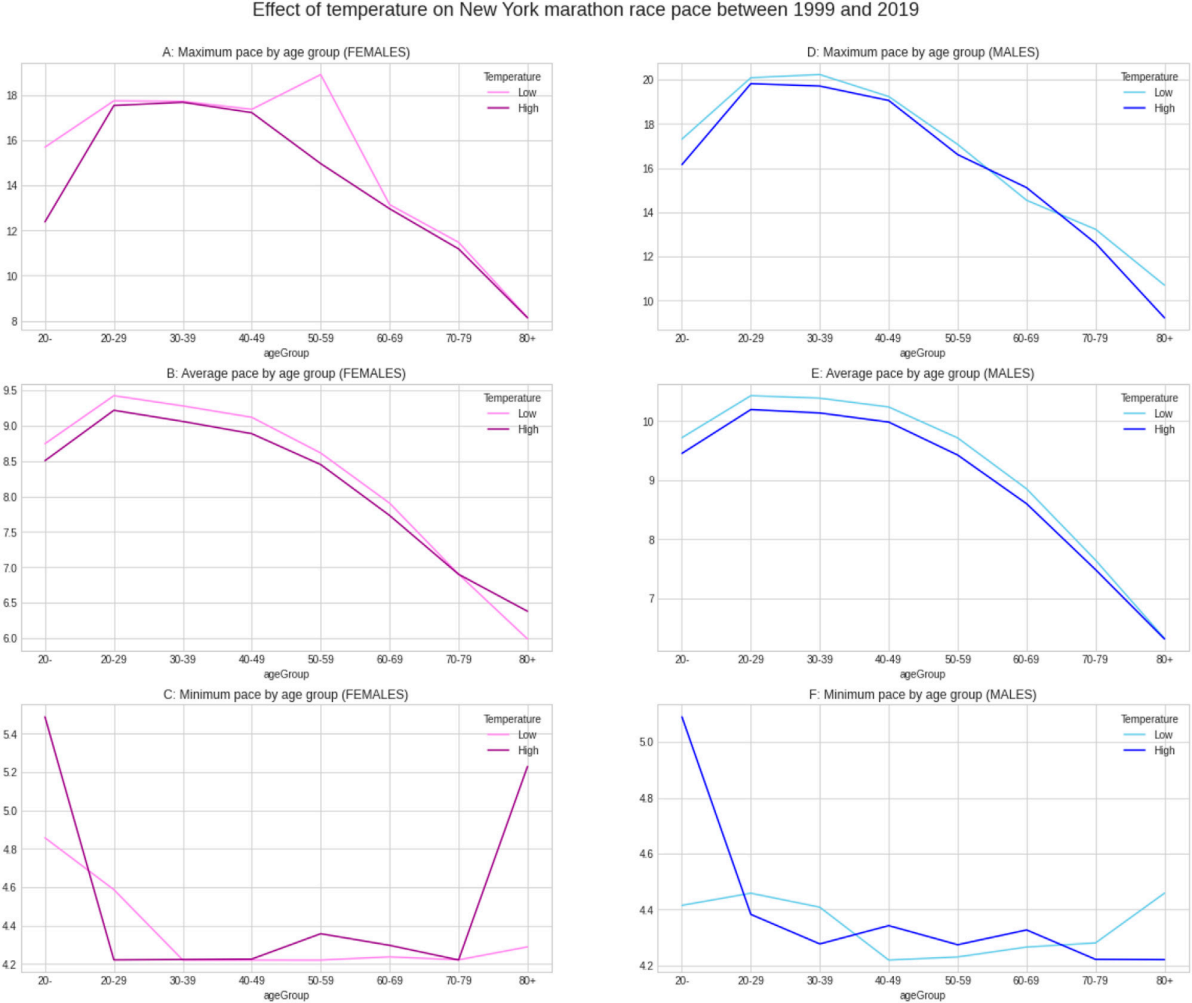
Effect of temperature on running speed by age groups.

**FIGURE 12:**
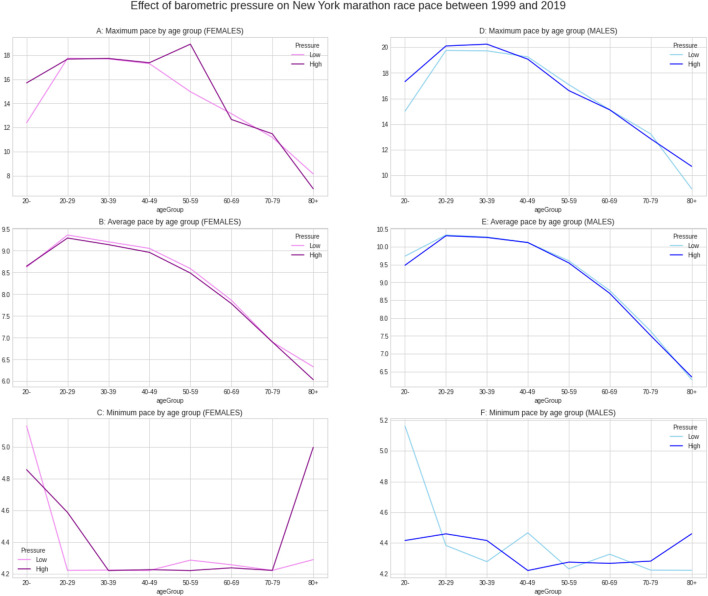
Effect of barometric pressure on running speed by age groups.

**FIGURE 13:**
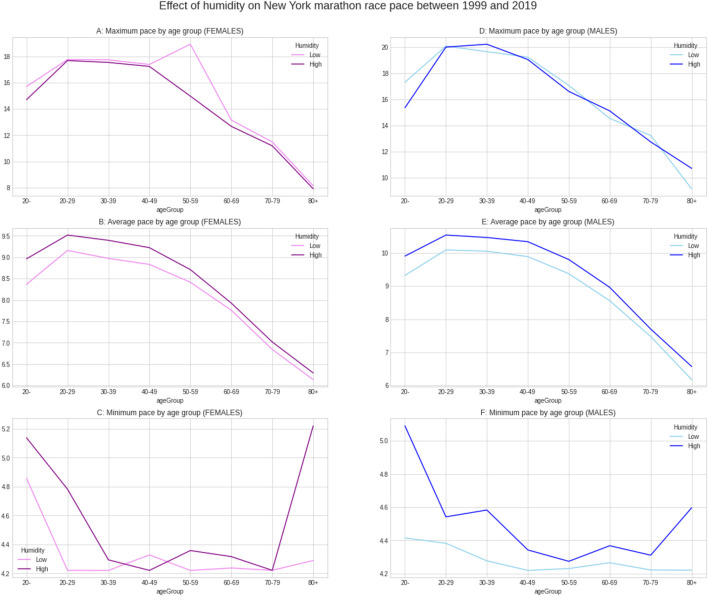
Effect of humidity on running speed by age groups.

**FIGURE 14:**
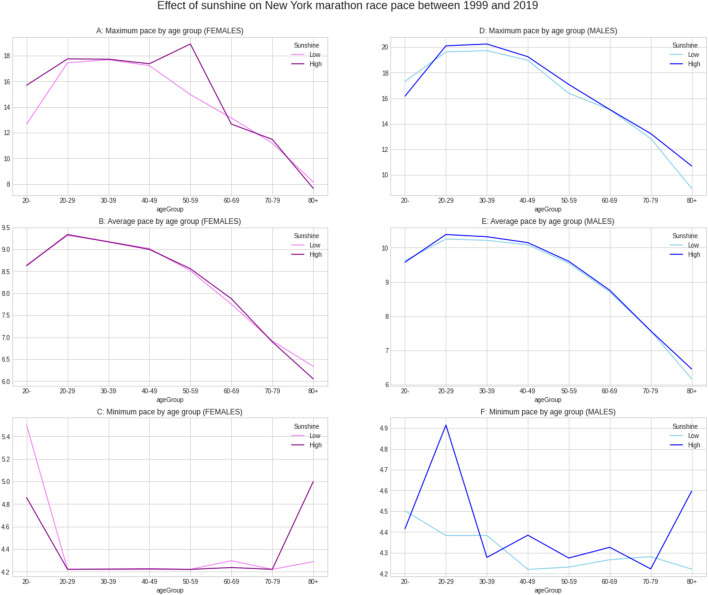
Effect of sunshine on running speed by age groups.

Figure 5 shows the weather conditions for the years 1999–2019. The average temperature was ∼9°C, average barometric pressure ∼1,020 hPa, average humidity ∼64% and sunshine duration ∼43 min per hour (Table 1). There was a positive correlation between the barometric pressure and calendar year (r = 0.16) meaning that barometric pressure has been going up slightly with time (Figure 6). Figure 7 presents the correlation matrix of running speed with average weather conditions from 09:00 a.m. to 04:00 p.m. with virtually no correlation of the weather conditions with running speed. When each age group was considered (Figure 8), again no correlation of weather conditions with running speed was found, with the exception of a weak but statistically significant Spearman correlation (ρ = 0.13) between running speed and humidity for runners of 80 years and older. The correlation analysis using time-adjusted average of weather conditions showed statistically weak Spearman correlations between running speed and humidity (*ρ* = 0.12) and temperature (*ρ* = -0.1) (Figure 9). The correlation analyses using time-adjusted average of weather conditions by age groups (Figure 10) showed significantly positive Spearman correlations between running speed and humidity for several age groups with the under 20 years showing the largest coefficient (*ρ* = 0.16). Similarly, in respect of temperature, in age groups 20–59 years, being the largest part of the dataset, a weak Spearman correlation coefficient (*ρ* = −0.11) was observed. In plotting and comparing the running speed values by age group for the different “high/low” weather categories, we found results that were consistent with the correlation analysis. Regarding temperature (Figure 11), the average pace charts (B and E, in the middle) are the most revealing and show that the “low” temperatures curves of pace stand above the “high” temperature curves, indicating an association of a faster running speed with lower temperatures. For barometric pressure (Figure 12), the average pace curves for “low” and “high” pressure stay close together, suggesting the different levels of barometric pressure and the average pace by age group were not statistically related in any way. Considering humidity (Figure 13), charts B and E (average pace) and also C and F (minimum pace) show that, in general, higher levels of humidity are associated with higher paces. This result was statistically significant. For sunshine (Figure 14), the curves of pace for high or low levels of sunshine stay close together for the average case (charts B and E) suggesting no relationship between them, whilst for the fastest and slowest cases, they seem to point at a very weak association of higher paces when sunshine levels are higher, to different degrees for the different age groups.

**TABLE 1:** Mean values with minimum, maximum, standard deviation and CI for hourly weather data. Minutes of sunshine are minutes of a full hour.

	Temperature (°C)	Barometric Pressure (hPa)	Humidity (%)	Sunshine (min)
mean	8.70	1,020.69	64.27	42.84
std	3.57	7.66	11.36	25.17
min	2	1,009	40	0
25%	6	1,014.75	57	15
50%	8	1,020	63	60
75%	12	1,025	70	60
max	17	1,035	97	60

## Discussion

This study intended to investigate a potential association between environmental conditions (i.e., temperature, humidity, barometric pressure and sunshine duration), and pacing for age group marathoners competing in the “New York City Marathon”. We have hypothesized that increasing temperatures during race day would have a higher impact on slower and older than on faster and younger runners. The main findings were i) fastest runners are those between 20 and 49 years; (ii) a positive correlation between humidity and running speed for runners across all age groups and iii) a negative correlation between temperature and running speed for middle-aged runners from 20 to 59 years iv) negative effects of high temperature had an increased effect on race times in 30–64 years old men and 40–64 years old women.

### The Influence of Humidity on Running Speed

A first important finding was that higher levels of humidity were associated with higher running speeds. The influence of humidity on running speed seemed, however, to depend upon age. We found positive correlations between humidity and running speed for age group runners. Few studies included humidity as an environmental parameter in their marathon running performance analyses (Vihma, 2010; El Helou et al., 2012; Maffetone et al., 2017; Mantzios et al., 2021). An analysis of the results of three European (i.e., Paris, London, Berlin) and three American marathon races (i.e., Boston, Chicago, New York) from 2001 to 2010 including 1,791,972 finishers and considering the influence of temperature, humidity, dew point and atmospheric pressure on marathon running performance showed no impact of humidity on performance (El Helou et al., 2012). A study on the influence of air temperature, relative and specific humidity, wind speed, solar shortwave radiation, thermal longwave radiation, and rain on the performance of finishers in the “Stockholm Marathon” between 1980 and 2008 showed that relative humidity was correlated with marathon race times. However, the humidity was significantly and negatively related to air temperature so the influence of humidity was due to air temperature (Vihma, 2010). A very recent study including all 1,280,557 finishers of the ‘New York City Marathon’ from the years 1970–2019 showed a negative association between humidity and race time where performance was lower on race days with low humidity (Knechtle et al., 2021). The influence was more pronounced for middle-aged runners where the effect of high humidity on race times was significantly increased in 40–59 years old men and 25–65 years old women (Knechtle, McGrath et al., 2021). An analysis from 1,258 marathon races, 50 km race-walk, 20 km race-walk, 10,000 m, 5,000 m and 3,000 m-steeplechase held between 1936 and 2019 in 42 countries showed that air temperature was the most predictive weather variable and humidity showed no influence (Mantzios et al., 2021). Considering both our findings and existing literature, we assume that humidity has only a minor influence on marathon running performance. Future studies might investigate the influence of humidity in a marathon race held in a tropical climate with high humidity and higher temperature.

### The Influence of Temperature in Running Speed

A second finding was that running speed decreased with increasing temperature supporting the idea of slower running speeds with higher temperatures. This influence was, however, dependent upon the age where we found a weak but statistically significant correlation in athletes of age groups 20–59 years. Despite the weak relationship among the temperature and running speed, the influence of higher temperatures on slower runners has been described in several studies (Ely et al., 2007; Ely et al., 2008; Vihma, 2010; El Helou et al., 2012; Nikolaidis et al., 2019; Knechtle et al., 2021; Knechtle et al., 2021). Factors associated with these results are most probably due to the slower running speed from start to finish of the slower runners rather than the decline in running speed (positive pacing) of faster runners (Ely, Martin et al., 2008). Besides that, evidence highlighted the relevance of surface area and mass to heat gain/loss in hot environments (Epstein et al., 1983). In this sense, runners with the highest body mass can be performance-limited regarding heat retention. Considering that, slower runners tend to present the highest values for body mass (especially, body fat), these morphological characteristics can be related to performance decrease among the non-professional runners (Thuany et al., 2021). An analysis from the “Berlin Marathon” with 882,540 finishers from 1974–2019 showed that the general mass of runners slowed down with increasing temperature and sunshine duration (Knechtle et al., 2021). A very recent study including all 1,280,557 finishers the “New York City Marathon” between 1970 and 2019 also showed a positive association between air temperature and race times (Knechtle et al., 2021). Also, the influence was pronounced for middle-aged runners where high temperatures had an increased effect on race times in 30–64 years old men and 40–64 years old women (Knechtle et al., 2021). Interestingly, elite marathoners (world record runners, top three and top ten finishers) improved performance with increasing temperature (Knechtle et al., 2021; Knechtle et al., 2021), what can be associated with better acclimatization for heat environment.

### Limitations, Strengths and Implications for Future Research

Limitations of this study include the lack of information regarding athletes (i.e., nutrition, hydration during the race, runners’ experience, previous strategy) and environmental characteristics (i.e., altimetry changes along the race, hydration stations). A further limitation is that we have no data about food (Burke, 2007) and fluid (Hew-Butler et al., 2006) intake during the marathon. Both fluid overload and hyponatremia (Mettler et al., 2008) and dehydration (Knechtle et al., 2011) might impair marathon running performance. Based on the limited data we cannot speculate on the relation between the hydrations status and pacing strategy of the runners. However, dehydration and body mass loss seemed not to impair marathon running performance (Zouhal et al., 2011; Berger et al., 2021) although past studies have shown a decreased ability of self-pacing when dehydrated (Stearns et al., 2009).

In this sense, we suggest that generalization should be done carefully. Nonetheless, the present study can be used by coaches, stakeholders and runners to improve pre-race preparation and optimize race performance. Athletes between 20 and 49 years present the best performance and are most negatively influenced by the temperature increase. The negative effects of high temperatures increased race times in 30–64 years old men and 40–64 years old women. Future studies might investigate the influence of humidity in a marathon race held in a tropical climate with high humidity and higher temperature. Likewise, it would be interesting to investigate if hydration provided by marathon organization can decrease the negative influence of environmental conditions on runners’ performance.

## Conclusions

In runners competing in the “New York City Marathon” between 1999 and 2019, temperature and humidity affected pacing in age group marathoners differently. The increasing temperature slowed runners of 20–59 years old with a pronounced negative effect for men aged 30–64 years and women aged 40–64 years. Increasing humidity slowed runners of all age groups and sexes. Practical implications include the consideration of acclimation for runners who need to improve performance.

## Data Availability

The raw data supporting the conclusion of this article will be made available by the authors, without undue reservation.
